# An Anonymous User Authentication and Key Agreement Scheme Based on a Symmetric Cryptosystem in Wireless Sensor Networks

**DOI:** 10.3390/s16081299

**Published:** 2016-08-16

**Authors:** Jaewook Jung, Jiye Kim, Younsung Choi, Dongho Won

**Affiliations:** 1 Department of Computer Engineering, Sungkyunkwan University, 2066 Seoburo, Suwon, Gyeonggido 440-746, Korea; jwjung@security.re.kr (J.J.); jykim@security.re.kr (J.K.); 2 Department of Cyber Security, Howon University, 64 Howon University 3 Gil, Impi-Myeon, Gunsan-Si, Jeonrabuk-Do 54058, Korea; yschoi@howon.ac.kr

**Keywords:** wireless sensor networks, mutual authentication, key agreement, BAN-logic, smart card

## Abstract

In wireless sensor networks (WSNs), a registered user can login to the network and use a user authentication protocol to access data collected from the sensor nodes. Since WSNs are typically deployed in unattended environments and sensor nodes have limited resources, many researchers have made considerable efforts to design a secure and efficient user authentication process. Recently, Chen et al. proposed a secure user authentication scheme using symmetric key techniques for WSNs. They claim that their scheme assures high efficiency and security against different types of attacks. After careful analysis, however, we find that Chen et al.’s scheme is still vulnerable to smart card loss attack and is susceptible to denial of service attack, since it is invalid for verification to simply compare an entered ID and a stored ID in smart card. In addition, we also observe that their scheme cannot preserve user anonymity. Furthermore, their scheme cannot quickly detect an incorrect password during login phase, and this flaw wastes both communication and computational overheads. In this paper, we describe how these attacks work, and propose an enhanced anonymous user authentication and key agreement scheme based on a symmetric cryptosystem in WSNs to address all of the aforementioned vulnerabilities in Chen et al.’s scheme. Our analysis shows that the proposed scheme improves the level of security, and is also more efficient relative to other related schemes.

## 1. Introduction

Wireless sensor networks (WSNs) are progressive ad hoc networks that are composed of quite a lot of resource-constrained sensor nodes that are randomly deployed over the target region [1]. Such networks provide cost-effective keys to a scope of monitoring problems, such as military battlefields, health care services, smart grid networks, and ubiquitous computing environments [2]. Moreover, the advanced technologies in the field of WSNs that a sensor attached to a device communicates with other ambient sensors are enabling to open the IoT environment. For these reasons, WSNs have been widely studied, both in the academic and industrial fields.

In WSNs, data gathered from sensor nodes sometimes include valuable and classified information such as details of the environmental surroundings during wartime, patient’s private information, monitoring information of museums, and the voltage variation monitoring data in electric power companies. In order to ensure the confidentiality and reliability of deployed WSNs, it is important that access be allowed to registered and legitimate users only. In addition, secure protocol construction positively requires a mutual authentication between an user and a sensor node. That is to say, a sensor node should be able to verify transmitted packet from a user to test a user’s legitimacy. Meanwhile, a user also should be able to verify transmitted packet from a sensor node to test a normality of the sensor node. Besides, because of resource-constrained characteristics such as limited power, communication and computational capabilities [3], the mutual authentication and key agreement protocol should not be complex and resource consuming. For example, an asymmetric key cryptosystem, like RSA [4,5], ECC [6] or El-gamal [7,8], requires a high computational overhead that is unsuitable for the energy constraints of WSNs. Therefore, the authentication and key agreement protocols for WSNs should be designed to consider both security and efficiency perspectives.

### 1.1. Related Studies

In 1981, Lamport [9] first proposed a remote password authentication protocol for insecure channels, and since then, many authentication protocols have been studied [10,11,12,13,14,15,16,17,18,19,20,21,22,23,24], in order to enhance security and efficiency. In 2006, Wong et al. [10] proposed a password-based user authentication scheme with a light computational overhead using a one-way hash function and exclusive-OR operations. However, Tseng et al. [11] pointed out that Wong et al.’s scheme [10] could not resist replay and forgery attacks, and then proposed an enhanced scheme. They claimed that their scheme was secure against reply and forgery attacks, and that it provided improved efficiency in the password change process. In 2009, Vaidya et al. [12] described how neither the schemes provided by Wong et al. [10] and Tseng et al. [11] were secure against replay attacks and man-in-the-middle attacks. They also proposed a robust user authentication scheme for the WSN environment. In the same year, Das [13] proposed an enhanced authentication scheme as the basis for Wong et al.’s scheme [10]. He insisted that their scheme can resist different types of attacks, such as many logged-in-users with the same login identity attacks, off-line password guessing attacks, stolen-verifier attacks and impersonation attacks. However, Khan and Alghathbar [14] pointed out in 2010 that Das’s scheme [13] could not resist gateway node bypassing attacks and privileged-insider attacks, and thus proposed an improved authentication scheme. In the same year, Vaidya et al. [15] demonstrated that Das’s scheme [13] and Khan-Alghathbar’s scheme [14] could not resist stolen smart card attacks, and they then proposed a enhanced version. Chen and Shih [16] also pointed out that Das scheme [13] could not resist parallel session attacks, and did not provide mutual authentication. In 2011, Fan et al. [17] proposed a user authentication protocol for two-tiered wireless sensor networks, and Yeh et al. [18] proposed an authentication protocol based on elliptic curves cryptography. In 2012, Das et al. [19] and Xue et al. [20] proposed a user authentication and key agreement scheme for WSNs based on the use of a smart card. These were both designed to fulfill various security requirements, such as key agreement, mutual authentication, password protection and prevention against several attacks. In 2014, Yuan [21] proposed an user authentication scheme based on biometric technique for WSNs. In the same year, Turkanović et al. [22] proposed a hash function based user authentication and key agreement protocol for heterogeneous ad hoc WSNs. They claimed that their scheme ensures a secure key agreement and mutual authentication and that it is also resilient against different types of attacks. However, Farash et al. [23] pointed out some security flaws in Turkanović et al.’s scheme [22], including a vulnerability to stolen-smart card attacks, man-in-the-middle attacks and sensor node impersonation attacks as well as the disclosure of secret parameters and the session key. They also suggested a user authentication and key agreement scheme for heterogeneous WSNs tailored for IoT environments. Recently, Amin et al. [24] demonstrated that Farash et al.’s scheme [23] could not resist stolen-smart card attacks, off-line password guessing attacks, user impersonation attacks, and known session-specific temporary information attacks, and proposed a improved version. Additionally, two-way authentication solutions on constraint devices using Datagram Transport Layer Security (DTLS) and Bellare-Canetti-Krawczyk (BCK) are proposed [25,26]. Porambage et al. [27] proposed an ECC-based authentication and key establishment scheme for WSNs in distributed IoT applications.

### 1.2. Motivations and Contributions

Chen et al. [28] recently suggested a secure user authentication scheme for wireless sensor networks. They claimed that their scheme could withstand different types of attacks, such as smart card loss attacks [29], replay attacks [30], stolen verifier attacks [31], privileged-insider attacks [32], user impersonation attacks [33], password guessing attacks [34], etc. They also claimed that their scheme was highly efficient, and very suited to WSN environments.

After performing a security analysis of Chen et al.’s scheme [28], however, we find that their scheme is still vulnerable to smart card loss attack, and is susceptible to denial of service attack, because it uses the incorrect verification method. In addition, we observe that their scheme cannot preserve user anonymity because user’s identity is in plaintext form in login request message. Furthermore, their scheme cannot quickly detect an incorrect password during login phase, and this flaw wastes both communication and computational overheads.

In this paper, we describe how these attacks work, and propose an anonymous two-factor user authentication and key agreement scheme based on a symmetric cryptosystem in WSNs to address all of the previously mentioned problems regarding Chen et al.’s scheme [28].

### 1.3. The Threat Model

This subsection describes the threat model that we constructed with some common assumptions, including the capabilities of an attacker in WSNs environment.

(1) An attacker can control the communication channels between the user, gateway node, and sensor node, meaning that the attacker can intercept or modify any messages that are transmitted via the public channel [35,36].
(2) An attacker can modify and resend the intercepted/eavesdropped message [37].
(3) All of the existing smart cards are vulnerable, because the confidential information that is stored within them can be extracted by physically monitoring the power consumption [38], meaning that an attacker could read the data that is stored on a smart card.
(4) Due to the hostile environments in the deployment field, sensor nodes can be physically captured by an attacker. However, the gateway node is secure, meaning that an attacker cannot obtain the parameters from the gateway node [18,19].
(5) An attacker can easily guess low-entropy passwords and identities in an off-line manner, but the guessing of two secret parameters (e.g., password, identity) is computationally infeasible in polynomial time [39].

### 1.4. Security Requirements for User Authentication Scheme

A secure and efficient password-based user authentication scheme should fulfill some security requirements and defend some different types of attacks. In this subsection, we will examine the essential requirements of authentication scheme based on previous researches [9,10,11,12,13,14,15,16,17,18,19,20,21,22,23,24,28]. These requirements will be used to analyze the security of our proposed scheme in Section 5.

(1) User anonymity: A user’s identity should be protected even if an attacker exploits user’s smart card used for authentication scheme or if the messages which exchanged in communication group are exposed.
(2) Mutual authentication: Mutual authentication should be carried out between the user and gateway node, the gateway node and sensor node, and the sensor node and user, respectively.
(3) Session key agreement: The session key should be securely shared among other communication parties after the verification procedure is finished.
(4) Quick detection of the incorrect password: If a user enters the incorrect password by mistake in login phase, the password should be detected before performing verification phase.
(5) User friendliness: This property allows users to freely change/update their password without needing to communicate with the gateway node.
(6) Robustness: User authentication schemes should withstand different types of attacks.
  - Smart card loss attacks: If an attacker steals a user’s smart card, the attacker can extract the contents by the power consumption technique [38]. With obtained information, the attacker can try to launch various types of attacks.
  - Off-line identity/password guessing attacks: An attacker tries to guess a identity/password and eventually find out the exact identity/password in an off-line environment by using the information stored in the smart card.
  - User impersonation attacks: An attacker pretends to be the registered user with the forged login message by using the secret or public information that is collected from the smart cards and the data packets.
  - Replay attacks: An attacker intercepts data packets for the purpose of making use of that data in some manner. Typically, this type of attack connotes copying and possibly modifying the data in various ways.
  - Privileged-insider attacks: A privileged-insider attack literally means the attack mounted by a malicious insider. The malicious insiders have a noticeable advantage over external attackers because they have an authorized system admission and also may be familiar with the network design and system actions. Commonly, the malicious insiders want to obtain the users’ private information such as their passwords.
  - Denial of Service (DoS) attacks: A DoS attack is any event that diminishes or eliminates a network’s capability of performing its expected function. In other words, an attacker mounts a DoS attack to make the server unavailable.
  - Stolen-verifier attacks: An attacker steals a password-verifier from the gateway node and directly use it to masquerade as a legitimate user.
  - Gateway node impersonation attacks: An attacker pretends to be the valid gateway node using the captured information.

### 1.5. Notations

All the notations mentioned in our proposed scheme and Chen et al.’s are specified in Table 1.

### 1.6. Organization of the Paper

The remainder of this paper is organized as follows: Section 2 reviews Chen et al.’s scheme, while Section 3 points out the weaknesses in Chen et al.’s scheme. Section 4 and Section 5 present the proposed scheme and the security analysis of the proposed scheme, respectively. Section 6 analyzes the performance of the proposed scheme in terms of the computational and communication costs; and lastly, Section 7 concludes the paper.

## 2. Review of Chen et al.’s Scheme

**Figure 1:**
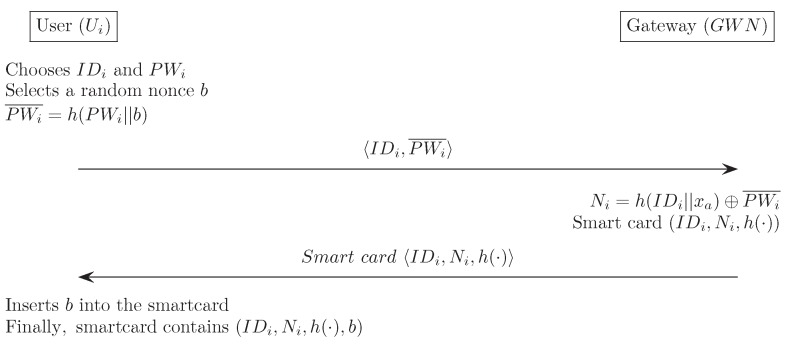
Registration phase for Chen et al.’s scheme.

**Figure 2:**
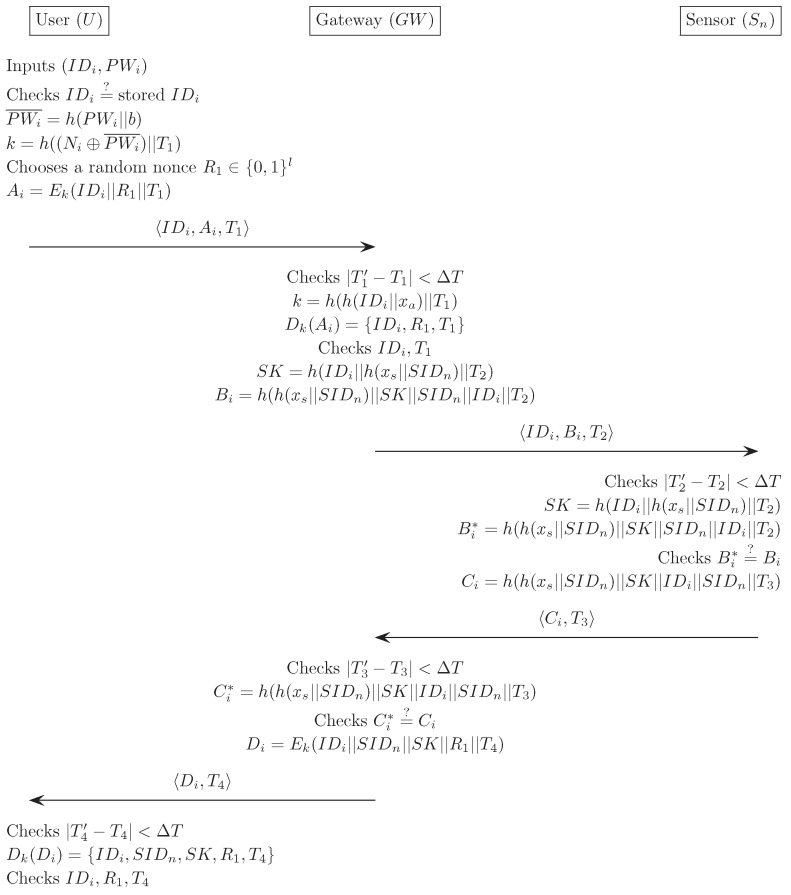
Login and verification phase for Chen et al.’s scheme.

**Figure 3:**
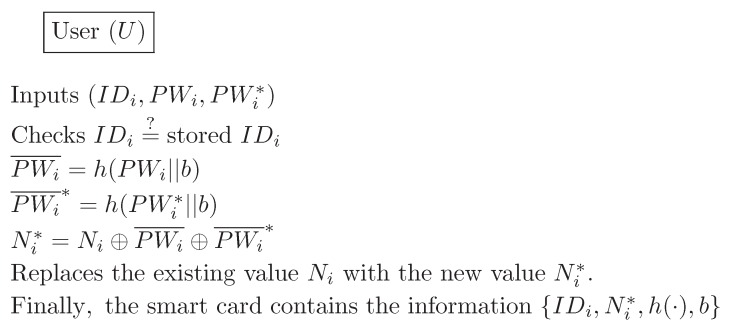
Password change phase for Chen et al.’s scheme.

In this section, we describe Chen et al.’s authentication scheme [28]. Three communication parties comprise a user $U_{i}$, a gateway node GWN, and a sensor node $S_{n}$. This scheme is composed of four phases: registration, login, verification, and password change. We describe each phase in detail, and Figure 1, Figure 2 and Figure 3 also illustrate Chen et al.’s scheme. Additionally, we describe the information on the sizes of all transmitted messages in the login and the verification phases. In order to compute the message size, based on [23], we set that both the block size of the symmetric encryption ($E_{k}$, $D_{k}$) and one-way hash function h(·) are 20 bytes long, the identity $ID_{i}$ and password $PW_{i}$ are 8 bytes, the random number *b* is 16 bytes, and the timestamp $T_{1}$–$T_{4}$ are 19 bytes long.

### 2.1. Registration Phase

(1) $U_{i}$ selects $ID_{i}$ and $PW_{i}$, and $U_{i}$ then generates a random nonce *b* that is only known to the $U_{i}$. $U_{i}$ computes a masked password $\bar{PW_{i}}=h(PW_{i}\left||b\right)$, and sends registration request message $\langle ID_{i},\bar{PW_{i}}\rangle$ to GWN through a secure channel.
(2) GWN computes $N_{i}=h(ID_{i}\left|\right|x_{a})⊕\bar{PW_{i}}$. GWN chooses a new smart card, and writes $\left\{ID_{i},N_{i},h\left(\cdot \right)\right\}$ into the smart card’s memory. Then, GWN sends the smart card to $U_{i}$ through a secure channel.
(3) $U_{i}$ enters the random nonce *b* in its memory. Finally, the smart card contains the information $\left\{ID_{i},N_{i},h\left(\cdot \right),b\right\}$.

### 2.2. Login Phase

(1) $U_{i}$ inserts $U_{i}$’s smart card into a terminal, and inputs the $ID_{i}$ and $PW_{i}$. The smart card compares $ID_{i}$ with the stored value $ID_{i}$. If this condition is satisfied, the smart card acknowledges the legitimacy of the $U_{i}$, and proceeds with the next step. Otherwise, it terminates this phase.
(2) The smart card computes $\bar{PW_{i}}=h(PW_{i}\left||b\right)$ and $k=h(\left(N_{i}⊕\bar{PW_{i}}\right)||T_{1})$, then chooses a random nonce $R_{1}\in {\left\{0,1\right\}}^{l}$, and computes $A_{i}=E_{k}(ID_{i}\left|\right|R_{1}\left|\right|T_{1})$.
(3) Finally, $U_{i}$ sends a login request message $\langle ID_{i},A_{i},T_{1}\rangle$ to GWN through a public channel.

From the above descriptions, in login phase of Chen et al.’s scheme, the message size of the login request $\langle ID_{i},A_{i},T_{1}\rangle$ can be computed as (8 + 20 + 19) = 47 bytes.

### 2.3. Verification Phase

(1) GWN first checks the validity of the time-stamp $|T_{1}^{\prime }-T_{1}|<\Delta T$. GWN computes $k=h(h(ID_{i}\left|\right|x_{a})||T_{1})$ and decrypts $D_{k}\left(A_{i}\right)=\left\{ID_{i},R_{1},T_{1}\right\}$. GWN then compares $ID_{i}$ and $T_{1}$ with the received values. If this condition is satisfied, GWN acknowledges the legitimacy of the $U_{i}$ and proceeds with the next step. Otherwise, it terminates this phase.
(2) GWN computes $SK=h(ID_{i}||h(x_{s}||SID_{n})||T_{2})$ and $B_{i}=h(h(x_{s}||SID_{n})||SK||SID_{n}||ID_{i}\left|\right|T_{2})$, then sends the message $\langle ID_{i},B_{i},T_{2}\rangle$ to $S_{n}$ through a public channel.
(3) $S_{n}$ checks whether $|T_{2}^{\prime }-T_{2}|<\Delta T$. $S_{n}$ then computes $SK=h(ID_{i}||h(x_{s}||SID_{n})||T_{2})$ and $B_{i}^{*}=h(h(x_{s}||SID_{n})||SK||SID_{n}||ID_{i}\left|\right|T_{2})$. $S_{n}$ compares $B_{i}^{*}$ with the received value $B_{i}$. If this condition is satisfied, $S_{n}$ believes that the GWN is authentic. Otherwise, it terminates this phase.
(4) $S_{n}$ computes $C_{i}=h(h(x_{s}||SID_{n})||SK||ID_{i}||SID_{n}\left|\right|T_{3})$, and then sends the message $\langle C_{i},T_{3}\rangle$ to GWN through a public channel.
(5) GWN checks whether $|T_{3}^{\prime }-T_{3}|<\Delta T$. GWN then computes $C_{i}^{*}=h(h(x_{s}||SID_{n})||SK||ID_{i}||SID_{n}\left|\right|T_{3})$, and compares it with the received value $C_{i}$. If true, GWN believes that the $S_{n}$ is authentic. Otherwise, GWN terminates this phase.
(6) GWN computes $D_{i}=E_{k}(ID_{i}||SID_{n}\left||SK|\right|R_{1}\left|\right|T_{4})$, and sends the message $\langle D_{i},T_{4}\rangle$ to $U_{i}$ through a public channel.
(7) $U_{i}$ checks whether $|T_{4}^{\prime }-T_{4}|\leq \Delta T$. $U_{i}$ decrypts $D_{k}\left(D_{i}\right)=\left\{ID_{i},SID_{n},SK,R_{1},T_{4}\right\}$ and compares $ID_{i}$, $R_{1}$ and $T_{4}$ with the previous values. If the verification does not hold, this phase is terminated. Otherwise, the $U_{i}$ believes that the GWN is authentic, and successfully ends the verification phase.

From the above descriptions, in verification phase of Chen et al.’s scheme, the message size of the $\langle ID_{i},B_{i},T_{2}\rangle$, $\langle C_{i},T_{3}\rangle$, and $\langle D_{i},T_{4}\rangle$ can be computed as (8 + 20 + 19) = 47 bytes, (20 + 19) = 39 bytes, and (20 + 19) = 39 bytes, respectively.

### 2.4. Password Change Phase

(1) $U_{i}$ inserts $U_{i}$’s smart card into a terminal and inputs $ID_{i}$, the old password $PW_{i}$ and new password $PW_{i}^{*}$. The smart card compares the entered value $ID_{i}$ with the $ID_{i}$ stored in the smart card. If this condition is not satisfied, it terminates this phase. Otherwise, the smart card proceeds with the next step.
(2) The smart card computes $\bar{PW_{i}}=h(PW_{i}\left||b\right)$, ${\bar{PW_{i}}}^{*}=h(PW_{i}^{*}\left||b\right)$ and $N_{i}^{*}=N_{i}⊕\bar{PW_{i}}⊕{\bar{PW_{i}}}^{*}$.
(3) The smart card replaces the existing value $N_{i}$ with the new value $N_{i}^{*}$. Finally, the smart card contains the information $\left\{ID_{i},N_{i}^{*},h\left(\cdot \right),b\right\}$.

## 3. Security Weaknesses of Chen et al.’s Scheme

In this section, we analyze the security of Chen et al.’s scheme [28]. Chen et al. claim that the scheme can withstand different types of attacks; however, based on attacker capabilities in Section 1.3, we found that their scheme is still vulnerable to smart card loss attack, and is also susceptible to denial of service attack, because it uses the incorrect verification method. In addition, we found that their scheme cannot preserve user anonymity. Since user’s identity included in a login request message is in plain-text form when it transmitted to GW-node in login phase. In detail, user’s identity on a public channel can be easily exposed to attackers, because they are able to eavesdrop on a public channel, as mentioned in Section 1.3. Furthermore, Chen et al.’s scheme missed a verification process to test input password, which led to the inefficiency problem. Since it is not able to detect an incorrect password during login phase, the login request message composed of incorrectly entered password sends to GW-node, and then GW-node detects the wrong message while performing a checking process on the login request message. Generally, the verification on the input password is recommended to perform immediately in login phase to avoid inefficiency problem [40]. We now describe the detailed weaknesses of Chen et al.’s schemes.

### 3.1. Smart Card Loss Attack

Suppose the smart card of $U_{i}$ is stolen by the attacker, who extracts the stored secret values $\left\{ID_{i},N_{i},h\left(\cdot \right),b\right\}$ through physically monitoring the power consumption [38] as described in Section 1.3. With this information, the attacker can successfully lead to following malicious scenarios.

Scenario 1: If the attacker obtains the smart card, he or she can easily expose a user’s identity $ID_{i}$ through physically monitoring the power consumption [38]. Disclosure of the user’s identity $ID_{i}$ may allow tracking of the $U_{i}$’s behavior and his or her current location.
Scenario 2: Using obtained smart card, the attacker can successfully pass the checking process of the login phase through using the $ID_{i}$ in the smart card, because their checking process just compares the entered $ID_{i}$ with the stored $ID_{i}$ in the smart card. The same situation also happens for the password change phase.

Therefore, Chen et al.’s scheme still suffers from smart card loss attack.

### 3.2. Denial of Service Attack

When the attacker steals the user’s smart card, the attacker can obtain the user’s identity $ID_{i}$ through physically monitoring the power consumption [38]. Through using this, in the password change phase, the attacker can easily set a new password, since it is invalid for verification to simply compare an entered $ID_{i}$ and a stored $ID_{i}$ in smart card. The following is a detailed description:

Step 1. The attacker inserts the $U_{i}$’s smart card into a terminal, and enters the $ID_{i}$, $PW_{a}$ and $PW_{a}^{*}$, where $PW_{a}$ and $PW_{a}^{*}$ are the attacker’s arbitrary new passwords.
Step 2. The smart card compares the entered value $ID_{i}$ with the $ID_{i}$ stored in the smart card. At this time, it is obvious that this verification process turns out to be successful, since the entered $ID_{i}$ is the same as the stored one in the smart card.
Step 3. The smart card computes $\bar{PW_{a}}=h(PW_{a}\left||b\right)$, ${\bar{PW_{a}}}^{*}=h(PW_{a}^{*}\left||b\right)$ and $N_{a}=N_{i}⊕\bar{PW_{a}}⊕{\bar{PW_{a}}}^{*}$.
Step 4. The smart card successfully replaces $N_{i}$ with the new value $N_{a}$.

If an attacker stole the $U_{i}$’s smart card and changed the password to an arbitrary new password as described above steps, then succeeding login requests by the legal user $U_{i}$ will be rejected, unless they re-register with the GWN again. Therefore, Chen et al.’s scheme is vulnerable to a denial of service attack.

### 3.3. Failure to Preserve User Anonymity

User anonymity is a highly desirable requirement for user authentication schemes, because of the leakage of user’s identity may allow an unauthorized entity to track the user’s login record and behavior pattern. However, Chen et al.’s scheme states that a user’s identity $ID_{i}$ is in plaintext form during the login and verification phase. As described in Section 1.3, using an eavesdropping attack, the attacker can maliciously monitor the public channels [35,36], and also identify some of the valuable information in messages transmitted over these public channels.

In this manner, an attacker can without difficulty eavesdrop on login messages to collect the plaintext identities of communicating users. All of the eavesdropped messages can be analyzed by the attacker to track down the connections among the $U_{i}$, GWN and $S_{n}$, and for this reason, user anonymity cannot be preserved in Chen et al.’s proposal [28].

### 3.4. Incorrect Password Cannot be Quickly Detected

During the login phase of Chen et al.’s scheme [28], if the $U_{i}$ inputs his/her identity and password, the smart card does not verify the validity of the $U_{i}$’s password; therefore, if the $U_{i}$ inputs an incorrect password by mistake, the login and verification phases are still carried out until they have been checked by GWN, leading to unnecessary communication and computational costs. The following detailed scenario explains this further.

Assume that the $U_{i}$ inputs the $ID_{i}$ and incorrect password $PW_{i}^{*}$ during the login phase; the smart card then computes the following:

$$\begin{array}{ccc} {\bar{PW_{i}}}^{*} & = & h(PW_{i}^{*}||b)\\ k^{*} & = & h(\left(N_{i}⊕{\bar{PW_{i}}}^{*}\right)||T_{1})\\ R_{1} & \in & {\left\{0,1\right\}}^{l}\\ A_{i}^{*} & = & E_{k^{*}}(ID_{i}\left|\right|R_{1}\left|\right|T_{1}) \end{array}$$

$U_{i}$ sends a login request message $\langle ID_{i},A_{i}^{*},T_{1}\rangle$ to GWN through a public channel. After receiving the login request message, GWN checks the validity of the time-stamp $|T_{1}^{\prime }-T_{1}|<\Delta T$. GWN computes $k=h(h(ID_{i}\left|\right|x_{a})||T_{1})$ and tries to decrypt $D_{k}\left(A_{i}^{*}\right)=\left\{ID_{i},R_{1},T_{1}\right\}$. GWN then compares $ID_{i}$ and $T_{1}$ with the received values. If this comparison is satisfied, the GWN believes that the $U_{i}$ is authentic. If not, it rejects the login request. However, it is obvious that GWN cannot decrypt $D_{k}\left(A_{i}^{*}\right)$, since $k^{*}$ is not equal to *k*. Therefore, GWN belatedly realizes that entered password $PW_{i}^{*}$ is an incorrect value, and GWN then terminates this procedure.

## 4. The Proposed Scheme

In this section, we propose an anonymous two-factor user authentication and key agreement scheme based on a symmetric cryptosystem in WSNs that addresses the security vulnerabilities in Chen et al.’s scheme [28]. Our proposed scheme also consists of the following four phases: registration, login, verification, and password change. We describe each phase in detail, and also describe the information on the sizes of all transmitted messages in the login and the verification phases. Table 1 summarizes the notation for the proposed scheme.

### 4.1. Registration Phase

**Figure 4:**
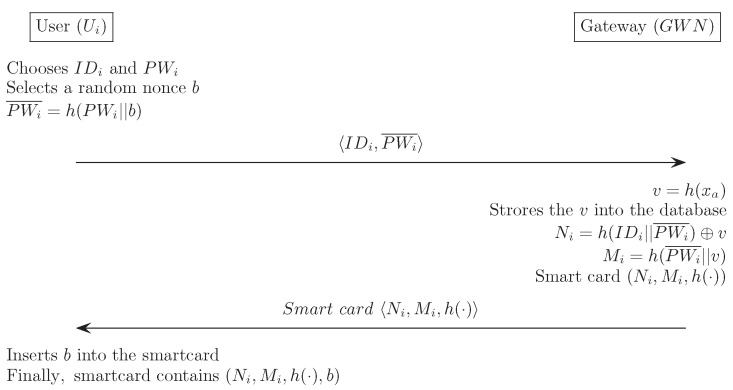
Registration phase for the proposed scheme.

The user registration phase begins when the $U_{i}$ sends a registration request with his/her identity and a hashed password to GWN. The GWN then issues a smart card that stores some information, and sends it to $U_{i}$ as a response to the registration request. The following describes this process in detail, and Figure 4 illustrates the registration phase for our proposed scheme.

(1) $U_{i}$ selects $ID_{i}$ and $PW_{i}$, and $U_{i}$ then generates a random nonce *b*, that is only known to the $U_{i}$. $U_{i}$ computes a masked password $\bar{PW_{i}}=h(PW_{i}\left||b\right)$ and sends registration request message $\langle ID_{i},\bar{PW_{i}}\rangle$ to GWN through a secure channel.
(2) GWN computes $v=h(x_{a})$, $N_{i}=h(ID_{i}\left|\right|\bar{PW_{i}})⊕v$ and $M_{i}=h(\bar{PW_{i}}\left||v\right)$, and stores the *v* into the database. GWN then chooses a new smart card and writes $\left\{N_{i},M_{i},h\left(\cdot \right)\right\}$ into the smart card memory. After that the GWN sends the smart card to $U_{i}$ through a secure channel.
(3) Upon receiving the smart card, $U_{i}$ enters the random nonce *b* in its memory. Finally, the smart card contains the information $\left\{N_{i},M_{i},h\left(\cdot \right),b\right\}$.

### 4.2. Login Phase

**Figure 5:**
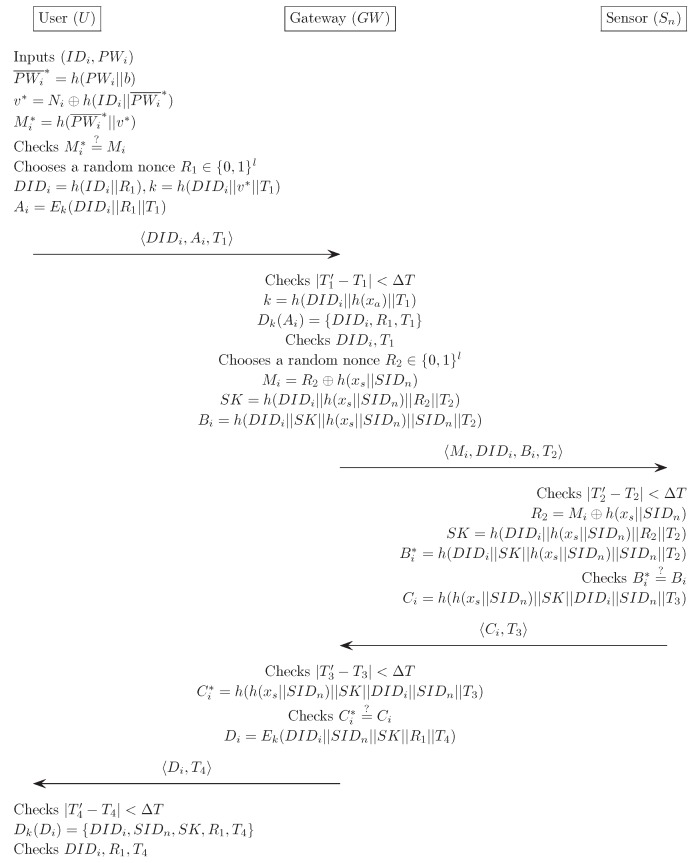
Login and verification phase for the proposed scheme.

The login phase is executed whenever the $U_{i}$ wants to gain access to WSN. In this phase, $U_{i}$ sends the login request to GWN. Figure 5 illustrates the login and verification phase for our proposed scheme. In detail, this process is:

(1) $U_{i}$ inserts $U_{i}$’s smart card into a terminal, and inputs the $ID_{i}$ and $PW_{i}$. The smart card computes the masked password ${\bar{PW_{i}}}^{*}=h(PW_{i}\left||b\right)$ and $v^{*}=N_{i}⊕h(ID_{i}\left|\right|{\bar{PW_{i}}}^{*})$. The smart card further computes $M_{i}^{*}=h({\bar{PW_{i}}}^{*}\left|\right|v^{*})$, and compares it with the stored value $M_{i}$. If this condition is satisfied, the smart card acknowledges the legitimacy of the $U_{i}$, and proceeds with the next step. Otherwise, it terminates this phase.
(2) The smart card chooses a random nonce $R_{1}\in {\left\{0,1\right\}}^{l}$, and computes $DID_{i}=h(ID_{i}\left|\right|R_{1})$. The smart card then computes $k=h(DID_{i}||v^{*}||T_{1})$ and $A_{i}=E_{k}(DID_{i}\left|\right|R_{1}\left|\right|T_{1})$.
(3) Finally, $U_{i}$ sends a login request message $\langle DID_{i},A_{i},T_{1}\rangle$ to GWN through a public channel.

From the above descriptions, in login phase of our propose scheme, the message size of the login request $\langle DID_{i},A_{i},T_{1}\rangle$ can be computed as (8 + 20 + 19) = 47 bytes.

### 4.3. Verification Phase

This phase executes several steps to achieve mutual authentication which is to test all transmitted message for judging the legitimacies of a $U_{i}$, GWN, and sensor node. As well as a session key agreement between all parties involved within the network. When GWN receives the login request message from the $U_{i}$, the verification phase begins. The following describes this process in detail.

(1) GWN first checks the validity of the time-stamp $|T_{1}^{\prime }-T_{1}|<\Delta T$. GWN computes $k=h(DID_{i}||h\left(x_{a}\right)||T_{1})$ and decrypts $D_{k}\left(A_{i}\right)=\left\{DID_{i},R_{1},T_{1}\right\}$. GWN then compares $DID_{i}$ and $T_{1}$ with the received values. If this condition is satisfied, GWN acknowledges the legitimacy of the $U_{i}$ and proceeds with the next step. Otherwise, it terminates this phase.
(2) GWN chooses $R_{2}\in {\left\{0,1\right\}}^{l}$, and computes $M_{i}=R_{2}⊕h(x_{s}||SID_{n})$. GWN further computes $SK=h(DID_{i}||h(x_{s}||SID_{n})||R_{2}\left|\right|T_{2})$ and $B_{i}=h(DID_{i}||SK||h(x_{s}||SID_{n})||SID_{n}\left|\right|T_{2})$, and then sends the message $\langle M_{i},DID_{i},B_{i},T_{2}\rangle$ to $S_{n}$ through a public channel.
(3) $S_{n}$ first checks whether $|T_{2}^{\prime }-T_{2}|<\Delta T$. If this condition does not hold, this phase is terminated. Otherwise, it computes $R_{2}=M_{i}⊕h(x_{s}||SID_{n})$ and $SK=h(DID_{i}||h(x_{s}||SID_{n})||R_{2}\left|\right|T_{2})$. The $S_{n}$ further computes $B_{i}^{*}=h(DID_{i}||SK||h(x_{s}||SID_{n})||SID_{n}\left|\right|T_{2})$ and compares it with the received value $B_{i}$. If this condition is satisfied, $S_{n}$ believes that the GWN is authentic. Otherwise, it terminates this phase.
(4) $S_{n}$ computes $C_{i}=h(h(x_{s}||SID_{n})||SK||DID_{i}||SID_{n}\left|\right|T_{3})$, and then sends the message $\langle C_{i},T_{3}\rangle$ to GWN through a public channel.
(5) GWN first checks whether $|T_{3}^{\prime }-T_{3}|<\Delta T$. If the relationship does not hold, this phase is terminated. Otherwise, it computes $C_{i}^{*}=h(h(x_{s}||SID_{n})||SK||DID_{i}||SID_{n}\left|\right|T_{3})$, and compares it with the received value $C_{i}$. If true, GWN believes that the $S_{n}$ is authentic. Otherwise, it terminates this phase.
(6) GWN computes $D_{i}=E_{k}(DID_{i}||SID_{n}\left||SK|\right|R_{1}\left|\right|T_{4})$, and sends the message $\langle D_{i},T_{4}\rangle$ to $U_{i}$ through a public channel.
(7) $U_{i}$ first checks whether $|T_{4}^{\prime }-T_{4}|\leq \Delta T$. If the relationship does not hold, it terminates this phase. Otherwise, it computes $D_{k}\left(D_{i}\right)=\left\{DID_{i},SID_{n},SK,R_{1},T_{4}\right\}$, and compares $DID_{i}$, $R_{1}$ and $T_{4}$ with the previous values. If the verification does not hold, it terminates this phase. Otherwise, the $U_{i}$ believes that GWN is authentic, and successfully ends the verification phase.

From the above descriptions, in verification phase of our proposed scheme, the message size of the $\langle M_{i},DID_{i},B_{i},T_{2}\rangle$, $\langle C_{i},T_{3}\rangle$, and $\langle D_{i},T_{4}\rangle$ can be computed as (20 + 20 + 20 + 19) = 79 bytes, (20 + 19) = 39 bytes, and (20 + 19) = 39 bytes, respectively.

### 4.4. Password Change Phase

**Figure 6:**
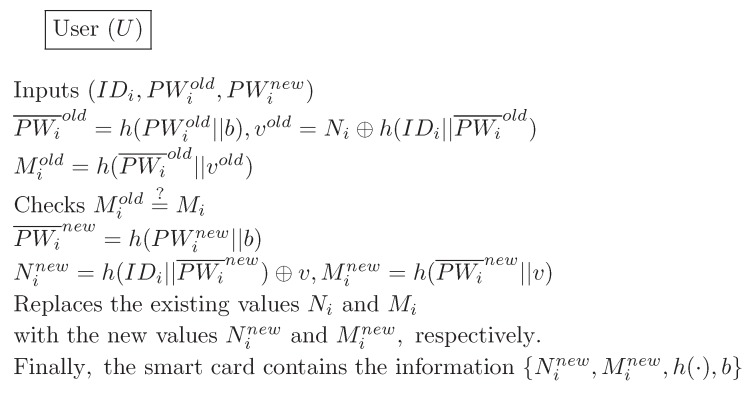
Password change phase for the proposed scheme.

The password change phase is invoked whenever the $U_{i}$ wants to change his or her old password to a new password. In the password change phase of our proposed scheme, $U_{i}$ communicates without any assistance from the GWN. Figure 6 illustrates the password change phase for our proposed scheme. We now describe this process in further detail:

(1) $U_{i}$ inserts $U_{i}$’s smart card into a terminal, and inputs $ID_{i}$, old password $PW_{i}^{old}$, and new password $PW_{i}^{new}$. The smart card computes the old masked password ${\bar{PW_{i}}}^{old}=h(PW_{i}^{old}\left||b\right)$, $v^{old}=N_{i}⊕h(ID_{i}\left|\right|{\bar{PW_{i}}}^{old})$, and $M_{i}^{old}=h({\bar{PW_{i}}}^{old}\left|\right|v^{old})$. The smart card then verifies whether $M_{i}=M_{i}^{old}$. If this condition is not satisfied, it terminates this phase. Otherwise, the smart card proceeds with the next step.
(2) The smart card computes ${\bar{PW_{i}}}^{new}=h(PW_{i}^{new}\left||b\right)$, $N_{i}^{new}=h(ID_{i}\left|\right|{\bar{PW_{i}}}^{new})⊕v$ and $M_{i}^{new}=h({\bar{PW_{i}}}^{new}\left||v\right)$
(3) The smart card replaces the existing values $N_{i}$ and $M_{i}$ with the new values $N_{i}^{new}$ and $M_{i}^{new}$, respectively. Finally, the smart card contains the information $\left\{N_{i}^{new},M_{i}^{new},h\left(\cdot \right),b\right\}$.

## 5. Security Analysis and Proof of the Proposed Scheme

In this section, we present a security analysis of our proposed scheme. We first examine whether our proposed scheme is safe, and we also consider its ability to resist various known attacks as described in Section 1.4. Then we adopt Burrows-Abadi-Needham (BAN) logic [41] to prove that a session key can be correctly generated between $U_{i}$, GWN and $S_{n}$.

### 5.1. Security Analysis of the Proposed Scheme

In this subsection, we scrutinize whether our proposed scheme can not only withstand various attacks, but also satisfy basic requirements that the security scheme claims. Moreover, we conduct a comparative analysis [13,14,15,16,17,18,19,20,28], which describes in Table 2. Details of the results are illustrated below.

**Proposition 1.**  The proposed scheme preserves user anonymity

**Proof.**  Suppose that the attacker has intercepted $U_{i}$’s login request message $\langle DID_{i},A_{i},T_{1}\rangle$. The attacker may then try to analyze the login request message by retrieving any static parameters from this message. However, it is not feasible to derive $ID_{i}$ from the login request message because the login request message includes $DID_{i}$ instead of $ID_{i}$. Thus the use of $DID_{i}$ ensures that the attacker cannot acquire any information related to the user identity. ☐

**Proposition 2.**  The proposed scheme achieves mutual authentication

**Proof.**  In our proposed scheme, the GWN can authenticate the user by checking whether the login request message is correct, and the $S_{n}$ can authenticate the GWN by checking whether the message $\langle M_{i},DID_{i},B_{i},T_{2}\rangle$ is correct. To authenticate the $S_{n}$, the GWN verifies whether the message $\langle C_{i},T_{3}\rangle$ received by the $S_{n}$ is valid or not. Also, the $U_{i}$ can authenticate the GWN by checking whether the message $\langle D_{i},T_{4}\rangle$ is correct. If all these verification processes are successfully finished, mutual authentication has been executed properly. ☐

**Proposition 3.**  The proposed scheme provides the session key agreement

**Proof.**  In our proposed scheme, the user and the sensor node can share the session key after the verification procedure. As a result of the randomness and independence of the generation of $R_{2}$ in all sessions, the shared session key $SK=h(DID_{i}||h(x_{s}||SID_{n})||R_{2}\left|\right|T_{2})$ differs for each session. Therefore, it is difficult for the attacker to compute the session key from the intercepted messages. ☐

**Proposition 4.**  The proposed scheme withstands smart card loss attacks

**Proof.**  Suppose smart card of $U_{i}$ is stolen by the attacker, who extracts secret values $\left\{N_{i},M_{i},h\left(\cdot \right),b\right\}$ through the studies [38]. Even if the attacker obtains $\left\{N_{i},M_{i},h\left(\cdot \right),b\right\}$, the attacker cannot know the user’s $ID_{i}$, because our proposed scheme does not allow the $ID_{i}$ to be stored in the smart card. In addition, as the ID in the smart card is erased, our proposed scheme uses a suitable password-based checking process, instead of a vulnerable id-based checking process. ☐

**Proposition 5.**  The proposed scheme withstands off-line password guessing attacks

**Proof.**  Suppose that the attacker extracts all of the secret information from the smart card. To successfully carry out a password guessing attack, the attacker has to know the $U_{i}$’s identity $ID_{i}$. However, in our proposed scheme, it is impossible for the attacker to obtain the $ID_{i}$. Furthermore, the guessing of two secret parameters (e.g., password, identity) is computationally infeasible in polynomial time. Thus, our proposed scheme is secure against off-line password guessing attacks. ☐

**Proposition 6.**  The proposed scheme withstands user impersonation attacks

**Proof.**  An attacker tries to impersonate a legal user $U_{i}$ in order to deceive other parties. To start a new session, the attacker has to modify the login request message $\langle DID_{i},A_{i},T_{1}\rangle$. In order to change these values, the attacker has to know the $ID_{i}$. However, there is no way to obtain the user’s $ID_{i}$. Therefore, our proposed scheme is secure against user impersonation attacks. ☐

**Proposition 7.**  The proposed scheme quickly detects the incorrect password

**Proof.**  In our proposed scheme, when the user inputs the incorrect password $PW_{a}$, the smart card calculates $\bar{PW_{a}}=h(PW_{a}\left||b\right)$ and $v_{a}=N_{i}⊕h(ID_{i}\left|\right|\bar{PW_{a}})$. The smart card further computes $M_{a}=h(\bar{PW_{a}}\left|\right|v_{a})$ and compares it with the stored value $M_{i}$. If this condition is satisfied, the card knows the user has entered the incorrect password. However, it is obvious that $M_{a}$ is not equal to $M_{i}$. Therefore, unlike Chen et al.’s scheme, the smart card can promptly detect the incorrect password at the beginning of the login phase. ☐

**Proposition 8.**  *The proposed scheme withstands replay attacks*

**Proof.**  An attacker can intercept data packets to make use of the data that is contained in some manner and can then try to login to the sensor node by using the intercepted packets that were transmitted between all parties involved. However, all messages transmitted in our proposed scheme include a current timestamp, such as $T_{1},T_{2},T_{3}$ or $T_{4}$. Hence, our proposed scheme can defend against replay attacks. ☐

**Proposition 9.**  The proposed scheme withstands privileged-insider attacks

**Proof.**  There is a possibility that a privileged insider can directly acquire the user’s password from the GWN to then access the user’s account in other systems by using the same password. This attack is a result of the disclosure of the user’s password during the registration phase. In our proposed scheme, the $U_{i}$ submits the password information to the GWN in the form of $\bar{PW_{i}}=h(PW_{i}\left||b\right)$, instead of the form $PW_{i}$. Accordingly, the privileged insider cannot acquire the user’s password as an attacker. ☐

**Proposition 10.**  The proposed scheme withstands denial of service attacks

**Proof.**  Suppose that the attacker obtains the user’s smart card, and extracts all of the information from the smart card. The attacker then tries to modify the password for denial of service attack. However, the attacker cannot change the password, because our proposed scheme uses a secure verification method at the beginning of the password change phase. To successfully pass this verification procedure, the attacker has to know the user $ID_{i}$ and $PW_{i}$. Therefore, our proposed scheme is secure for denial of service attack. ☐

**Proposition 11.**  The proposed scheme withstands stolen-verifier attacks

**Proof.**  An attacker acquires a password-verifier from the gateway node to immediately impersonate an authenticated user. To succeed in a stolen-verifier attack, the attacker needs to know the user’s password. However, as is shown in our proposed scheme, no verification table is stored in our proposed scheme. ☐

**Proposition 12.**  The proposed scheme withstands off-line identity guessing attacks

**Proof.**  Suppose that the attacker extracts all of the secret information from the smart card. To successfully carry out an off-line identity guessing attack, the attacker has to know user’s password $PW_{i}$. However, in our proposed scheme, the attacker cannot acquire the user’s password. Moreover, it is not feasible to obtain $ID_{i}$ from the login request because the login request includes $DID_{i}$ instead of $ID_{i}$. Therefore, the attacker does not know the user’s identity in our proposed scheme. ☐

**Proposition 13.**  *The proposed scheme provides a friendly and efficient password change phase*

**Proof.**  The ideal user authentication scheme allows the user to freely change his/her password, and this should be carried out without any assistance from other parties to ensure user friendliness and efficiency. In our proposed scheme, when the user wants to change an old password, the smart card first checks the validity of the old password $PW_{i}^{old}$. If the password is valid, the user can choose the new password $PW_{i}^{new}$, and the smart card computes the new values $N_{i}^{new}$ and $M_{i}^{new}$. Then smart card replaces the existing values with the new values. Thus, the password change phase for our proposed scheme is both user-friendly and effective because the user $U_{i}$ does not communicate with the gateway GWN. ☐

**Proposition 14.**  The proposed scheme withstands GW-node impersonation attacks

**Proof.**  Suppose that the attacker obtains all transmitted message such as $\langle DID_{i},A_{i},T_{1}\rangle$ and $\langle M_{i},DID_{i},B_{i},T_{2}\rangle$, and tries to impersonate as a legal gateway node. However, It is not feasible to decrypt the $A_{i}=E_{k}(DID_{i}\left|\right|R_{1}\left|\right|T_{1})$ without the symmetry key *k*. Therefore, the attacker can not impersonate as a valid gateway node. ☐

### 5.2. Authentication Proof with BAN Logic

We prove the way in which a session key can be correctly generated between communicating parties during the authentication process using a well-known formal logic known as BAN logic [41]; BAN logic is a formal means that is widely used to analyze the security of cryptographic protocols. The basic notation for figuring out BAN logic follows below.

- A◃S: The *A* sees the sentence *S*.
- A∣≡S: The sentence *S* is believed by *A*.
- ♯(S): It makes a fresh sentence *S*.
- A∣∼S: The *A* said the sentence *S*.
- $<S{>}_{K}$: Combine the sentence *S* using *K*.
- $A\overset{K}{⟷}B$: For secure communication, *A* and *B* share a secret key *K*.
- A⇒S: The sentence *S* is controled by *A*.
- ${\left\{S\right\}}_{K}$: Encrypt the sentence *S* using *K*
- ${\left(S\right)}_{K}$: Perform the hash operation to sentence *X* using *Y*.

Generally, BAN logic provides some rules as follows.

1. Message-meaning rule: $\frac{A|\equiv A\overset{K}{↔}B,A◃<S{>}_{K}}{A|\equiv B|∼S}$: If the key *K* is shared between *A* and *B*, *A* sees the *S* combined by *K*. Then *A* believes that *B* once said *S*.
2. Nonce-verification rule: $\frac{A|\equiv \#(S),A|\equiv B|∼S}{A|\equiv B|\equiv S}$: If *A* trusts that *S* is fresh and *A* believes *B* once said *S*, then *A* believes that *B* believes *S*.
3. The believe rule: $\frac{A|\equiv S,A|\equiv T}{A|\equiv (S,T)}$: If *S* and *T* are believed by *A*, then (S,T) are also believed by *A* .
4. Freshness-conjuncatenation rule: $\frac{A|\equiv \#(S)}{A|\equiv \#(S,T)}$: If freshness of *S* is believed by *A*, then *A* can trust the freshness of whole statement.
5. Jurisdiction rule: $\frac{A|\equiv B|\Rightarrow S,A|\equiv B|\equiv S}{A|\equiv S}$: If *A* establishes that *B* has jurisdiction over *S*, and *A* trusts that *B* trusts a statement *S*, then *A* also trusts *S*.

Our analysis based on BAN logic will fulfill the following goals:

- Goal 1. $U_{i}|\equiv \left(U_{i}\overset{SK}{⟷}GWN\right)$
- Goal 2. $U_{i}|\equiv GWN|\equiv \left(U_{i}\overset{SK}{⟷}GWN\right)$
- Goal 3. $GWN|\equiv (U_{i}\overset{SK}{⟷}GWN)$
- Goal 4. $GWN|\equiv U_{i}|\equiv \left(U_{i}\overset{SK}{⟷}GWN\right)$

Our message can be transformed into idealized form as follows:

- Message 1. $U_{i}\to S_{n}$: ${\left\{ID_{i},T_{1},U_{i}\overset{ID_{i}}{⟷}S_{n}\right\}}_{h(x_{a})}$
- Message 2. $U_{i}\to S_{n}$: ${\left\{ID_{i},R_{1},T_{1},U_{i}\overset{ID_{i}}{⟷}S_{n},U_{i}\overset{R_{1}}{⟷}S_{n}\right\}}_{h(x_{a})}$
- Message 3. $GWN\to S_{n}$: ${\left(SID_{n},T_{2},GWN\overset{SID_{n}}{⟷}S_{n}\right)}_{h(x_{s}||SID_{n})}$
- Message 4. $GWN\to S_{n}$: ${\left(SID_{n},R_{2},T_{2},GWN\overset{SID_{n}}{⟷}S_{n},GWN\overset{R_{2}}{⟷}S_{n}\right)}_{h(x_{s}||SID_{n})}$
- Message 5. $S_{n}\to GWN$: ${\left(T_{3},GWN\overset{SID_{n}}{⟷}S_{n},GWN\overset{R_{1}}{⟷}S_{n}\right)}_{h(x_{s}||SID_{n})}$
- Message 6. $U_{i}\to GWN$: ${\left(R_{1},T_{3},GWN\overset{SID_{n}}{⟷}S_{n},GWN\overset{R_{1}}{⟷}S_{n},U_{i}\overset{SK}{⟷}GWN\right)}_{h(x_{a})}$
- Message 7. $S_{n}\to U_{i}$: ${\left\{R_{2},T_{3},U_{i}\overset{ID_{i}}{⟷}S_{n},U_{i}\overset{R_{2}}{⟷}S_{n}\right\}}_{h(x_{a})}$
- Message 8. $GWN\to U_{i}$: ${\left(T_{4},R_{2},U_{i}\overset{ID_{i}}{⟷}S_{n},U_{i}\overset{h(x_{a})}{⟷}S_{n},U_{i}\overset{SK}{⟷}GWN\right)}_{h(x_{a})}$

We define some assumptions as follows, and these assumptions will be used in further proof.

- A1: $S_{n}|\equiv ♯\left(T_{1}\right)$
- A2: $S_{n}|\equiv ♯\left(T_{2}\right)$
- A3: $GWN|\equiv ♯(T_{3})$
- A4: $U_{i}|\equiv ♯\left(T_{3}\right)$
- A5: $U_{i}|\equiv ♯\left(T_{4}\right)$
- A6: $S_{n}|\equiv ♯\left(R_{1}\right)$
- A7: $S_{n}|\equiv ♯\left(R_{2}\right)$
- A8: $GWN|\equiv ♯(R_{1})$
- A9: $U_{i}|\equiv ♯\left(R_{2}\right)$
- A10: $U_{i}|\equiv \left(U_{i}\overset{h_{(}x_{a})}{⟷}S_{n}\right)$
- A11: $S_{n}|\equiv \left(U_{i}\overset{h_{(}x_{a})}{⟷}S_{n}\right)$
- A12: $S_{n}|\equiv \left(GWN\overset{h(x_{s}||SID_{n})}{⟷}S_{n}\right)$
- A13: $GWN|\equiv (GWN\overset{h_{(}x_{a})}{⟷}S_{n})$
- A14: $GWN|\equiv (GWN\overset{h(x_{s}||SID_{n})}{⟷}S_{n})$
- A15: $S_{n}|\equiv U_{i}\Rightarrow \left(U_{i}\overset{ID_{i}}{⟷}S_{n}\right)$
- A16: $S_{n}|\equiv U_{i}\Rightarrow \left(U_{i}\overset{R_{1}}{⟷}S_{n}\right)$
- A17: $S_{n}|\equiv GWN\Rightarrow \left(GWN\overset{SID_{n}}{⟷}S_{n}\right)$
- A18: $S_{n}|\equiv GWN\Rightarrow \left(GWN\overset{R_{2}}{⟷}S_{n}\right)$
- A19: $GWN|\equiv S_{n}\Rightarrow \left(GWN\overset{R_{1}}{⟷}S_{n}\right)$
- A20: $U_{i}|\equiv S_{n}\Rightarrow \left(U_{i}\overset{R_{2}}{⟷}S_{n}\right)$
- A21: $GWN|\equiv U_{i}\Rightarrow \left(U_{i}\overset{SK}{⟷}GWN\right)$
- A22: $U_{i}|\equiv GWN\Rightarrow \left(U_{i}\overset{SK}{⟷}GWN\right)$

Using the BAN logic rules, idealized form, and pre-defined some assumptions, we deploy our proof as follows:

Based on Message 1, we could derive:

S1 $S_{n}◃{\left\{ID_{i},T_{1},U_{i}\overset{ID_{i}}{⟷}S_{n}\right\}}_{h(x_{a})}$

According to the assumption A11 and the message meaning rule, we obtain:

S2 $S_{n}|\equiv U_{i}|∼\left(ID_{i},T_{1},U_{i}\overset{ID_{i}}{⟷}S_{n}\right)$

According to the assumption A1 and the freshness conjuncatenation rule, we obtain:

S3 $S_{n}|\equiv ♯\left(ID_{i},T_{1},U_{i}\overset{ID_{i}}{⟷}S_{n}\right)$

According to the S2, S3 and the nonce verification rule, we obtain:

S4 $S_{n}|\equiv U_{i}|\equiv \left(ID_{i},T_{1},U_{i}\overset{ID_{i}}{⟷}S_{n}\right)$

According to the S4 and the believe rule, we obtain:

S5 $S_{n}|\equiv U_{i}|\equiv \left(U_{i}\overset{ID_{i}}{⟷}S_{n}\right)$

According to the assumption A15 and the jurisdiction rule, we obtain:

S6 $S_{n}|\equiv \left(U_{i}\overset{ID_{i}}{⟷}S_{n}\right)$

According to the Message 2, we obtain:

S7 $S_{n}◃{\left\{ID_{i},R_{1},T_{1},U_{i}\overset{ID_{i}}{⟷}S_{n},U_{i}\overset{R_{1}}{⟷}S_{n}\right\}}_{h(x_{a})}$

According to the S7, assumption A11 and the message meaning rule, we obtain:

S8 $S_{n}|\equiv U_{i}|∼\left(ID_{i},R_{1},T_{1},U_{i}\overset{ID_{i}}{⟷}S_{n},U_{i}\overset{R_{1}}{⟷}S_{n}\right)$

According to the assumption A1, A6 and the freshness conjuncatenation rule, we obtain:

S9 $S_{n}|\equiv ♯\left(ID_{i},R_{1},T_{1},U_{i}\overset{ID_{i}}{⟷}S_{n},U_{i}\overset{R_{1}}{⟷}S_{n}\right)$

According to the S8, S9 and the nonce verification rule, we obtain:

S10 $S_{n}|\equiv U_{i}|\equiv \left(ID_{i},R_{1},T_{1},U_{i}\overset{ID_{i}}{⟷}S_{n},U_{i}\overset{R_{1}}{⟷}S_{n}\right)$

According to the S5, S6, S10 and the believe rule, we obtain:

S11 $S_{n}|\equiv U_{i}|\equiv \left(U_{i}\overset{R_{1}}{⟷}S_{n}\right)$

According to the assumption A16 and the jurisdiction rule, we obtain:

S12 $S_{n}|\equiv \left(U_{i}\overset{R_{1}}{⟷}S_{n}\right)$

According to the Message 3, we obtain:

S13 $S_{n}◃{\left(SID_{n},T_{2},GWN\overset{SID_{n}}{⟷}S_{n}\right)}_{h(x_{s}||SID_{n})}$

According to the S13, assumption A12 and the message meaning rule, we obtain:

S14 $S_{n}|\equiv GWN|∼\left(SID_{n},T_{2},GWN\overset{SID_{n}}{⟷}S_{n}\right)$

According to the assumption A2 and the freshness conjuncatenation rule, we obtain:

S15 $S_{n}|\equiv ♯\left(SID_{n},T_{2},GWN\overset{SID_{n}}{⟷}S_{n}\right)$

According to the S14, S15 and the nonce verification rule, we obtain:

S16 $S_{n}|\equiv GWN|\equiv \left(SID_{n},T_{2},GWN\overset{SID_{n}}{⟷}S_{n}\right)$

According to the S16 and the believe rule, we obtain:

S17 $S_{n}|\equiv GWN|\equiv \left(GWN\overset{SID_{n}}{⟷}S_{n}\right)$

According to the assumption A17 and the jurisdiction rule, we obtain:

S18 $S_{n}|\equiv \left(GWN\overset{SID_{n}}{⟷}S_{n}\right)$

According to the Message 4, we obtain:

S19 $S_{n}◃{\left(SID_{n},R_{2},T_{2},GWN\overset{SID_{n}}{⟷}S_{n},GWN\overset{R_{2}}{⟷}S_{n}\right)}_{h(x_{s}||SID_{n})}$

According to the S19, assumption A12 and the message meaning rule, we obtain:

S20 $S_{n}|\equiv GWN|∼\left(SID_{n},R_{2},T_{2},GWN\overset{SID_{n}}{⟷}S_{n},GWN\overset{R_{2}}{⟷}S_{n}\right)$

According to the assumption A2, A7 and the freshness conjuncatenation rule, we obtain:

S21 $S_{n}|\equiv ♯\left(SID_{n},R_{2},T_{2},GWN\overset{SID_{n}}{⟷}S_{n},GWN\overset{R_{2}}{⟷}S_{n}\right)$

According to the S20, S21 and the nonce verification rule, we obtain:

S22 $S_{n}|\equiv GWN|\equiv \left(SID_{n},R_{2},T_{2},GWN\overset{SID_{n}}{⟷}S_{n},GWN\overset{R_{2}}{⟷}S_{n}\right)$

According to the S17, S18, S21 and the believe rule, we obtain:

S23 $S_{n}|\equiv GWN|\equiv \left(GWN\overset{R_{2}}{⟷}S_{n}\right)$

According to the assumption A18 and the jurisdiction rule, we obtain:

S24 $S_{n}|\equiv \left(GWN\overset{R_{2}}{⟷}S_{n}\right)$

According to the Message 5, we obtain:

S25 $GWN◃{\left(T_{3},GWN\overset{SID_{n}}{⟷}S_{n},GWN\overset{R_{1}}{⟷}S_{n}\right)}_{h(x_{s}||SID_{n})}$

According to the S25, assumption A14 and the message meaning rule, we obtain:

S26 $GWN|\equiv S_{n}|∼\left(T_{3},GWN\overset{SID_{n}}{⟷}S_{n},GWN\overset{R_{1}}{⟷}S_{n}\right)$

According to the assumption A3 and the freshness conjuncatenation rule, we obtain:

S27 $GWN|\equiv ♯(T_{3},GWN\overset{SID_{n}}{⟷}S_{n},GWN\overset{R_{1}}{⟷}S_{n})$

According to the S26, S27 and the nonce verification rule, we obtain:

S28 $GWN|\equiv S_{n}|\equiv \left(T_{3},GWN\overset{SID_{n}}{⟷}S_{n},GWN\overset{R_{1}}{⟷}S_{n}\right)$

According to the S17, S18, S28 and the believe rule, we obtain:

S29 $GWN|\equiv S_{n}|\equiv \left(GWN\overset{R_{1}}{⟷}S_{n}\right)$

According to the assumption A19 and the jurisdiction rule, we obtain:

S30 $GWN|\equiv (GWN\overset{R_{1}}{⟷}S_{n})$

According to the Message 6, we obtain:

S31 $GWN◃{\left(R_{1},T_{3},GWN\overset{SID_{n}}{⟷}S_{n},GWN\overset{R_{1}}{⟷}S_{n},U_{i}\overset{SK}{⟷}GWN\right)}_{h(x_{a})}$

According to the S31, assumption A13 and the message meaning rule, we obtain:

S32 $GWN|\equiv U_{i}|∼\left(R_{1},T_{3},GWN\overset{SID_{n}}{⟷}S_{n},GWN\overset{R_{1}}{⟷}S_{n},U_{i}\overset{SK}{⟷}GWN\right)$

According to the assumption A3, A8 and the freshness conjuncatenation rule, we obtain:

S33 $GWN|\equiv ♯(R_{1},T_{3},GWN\overset{SID_{n}}{⟷}S_{n},GWN\overset{R_{1}}{⟷}S_{n},U_{i}\overset{SK}{⟷}GWN)$

According to the S32, S33 and the nonce verification rule, we obtain:

S34 $GWN|\equiv U_{i}|\equiv \left(R_{1},T_{3},GWN\overset{SID_{n}}{⟷}S_{n},GWN\overset{R_{1}}{⟷}S_{n},U_{i}\overset{SK}{⟷}GWN\right)$

According to the S17, S18, S29, S34 and the believe rule, we obtain:

S35 $GWN|\equiv U_{i}|\equiv \left(U_{i}\overset{SK}{⟷}GWN\right)$ **(Goal 4.)**

According to the assumption A21 and the jurisdiction rule, we obtain:

S36 $GWN|\equiv (U_{i}\overset{SK}{⟷}GWN)$ **(Goal 3.)**

According to the Message 7, we obtain:

S37 $U_{i}◃{\left\{R_{2},T_{3},U_{i}\overset{ID_{i}}{⟷}S_{n},U_{i}\overset{R_{2}}{⟷}S_{n}\right\}}_{h(x_{a})}$

According to the S37, assumption A10 and the message meaning rule, we obtain:

S38 $U_{i}|\equiv S_{n}|∼\left(R_{2},T_{3},U_{i}\overset{ID_{i}}{⟷}S_{n},U_{i}\overset{R_{2}}{⟷}S_{n}\right)$

According to the assumption A4, A9 and the freshness conjuncatenation rule, we obtain:

S39 $U_{i}|\equiv ♯\left(R_{2},T_{3},U_{i}\overset{ID_{i}}{⟷}S_{n},U_{i}\overset{R_{2}}{⟷}S_{n}\right)$

According to the S38, S39 and the nonce verification rule, we obtain:

S40 $U_{i}|\equiv S_{n}|\equiv \left(R_{2},T_{3},U_{i}\overset{ID_{i}}{⟷}S_{n},U_{i}\overset{R_{2}}{⟷}S_{n}\right)$

According to the S5, S6, S39 and the believe rule, we obtain:

S41 $U_{i}|\equiv S_{n}|\equiv \left(U_{i}\overset{R_{2}}{⟷}S_{n}\right)$

According to the assumption A20, S41 and the jurisdiction rule, we obtain:

S42 $U_{i}|\equiv \left(U_{i}\overset{R_{2}}{⟷}S_{n}\right)$

According to the Message 8, we obtain:

S43 $U_{i}◃{\left(T_{4},R_{2},U_{i}\overset{ID_{i}}{⟷}S_{n},U_{i}\overset{h(x_{a})}{⟷}S_{n},U_{i}\overset{SK}{⟷}GWN\right)}_{h(x_{a})}$

According to the S43, assumption A10 and the message meaning rule, we obtain:

S44 $U_{i}|\equiv S_{j}|∼\left(T_{4},R_{2},U_{i}\overset{ID_{i}}{⟷}S_{n},U_{i}\overset{h(x_{a})}{⟷}S_{n},U_{i}\overset{SK}{⟷}GWN\right)$

According to the assumption A5, A9 and the freshness conjuncatenation rule, we obtain:

S45 $U_{i}|\equiv ♯\left(T_{4},R_{2},U_{i}\overset{ID_{i}}{⟷}S_{n},U_{i}\overset{h(x_{a})}{⟷}S_{n},U_{i}\overset{SK}{⟷}GWN\right)$

According to the S44, S45 and the nonce verification rule, we obtain:

S46 $U_{i}|\equiv GWN|\equiv \left(T_{4},R_{2},U_{i}\overset{ID_{i}}{⟷}S_{n},U_{i}\overset{h(x_{a})}{⟷}S_{n},U_{i}\overset{SK}{⟷}GWN\right)$

According to the S5, S6, S41, S46 and the believe rule, we obtain:

S47 $U_{i}|\equiv GWN|\equiv \left(U_{i}\overset{SK}{⟷}GWN\right)$ **(Goal 2.)**

According to the assumption A22 and the jurisdiction rule, we obtain:

S48 $U_{i}|\equiv \left(U_{i}\overset{SK}{⟷}GWN\right)$ **(Goal 1.)**

Based on (Goal 1–Goal 4), we can assure that our proposed scheme provides the mutual authentication and agreement of the session key SK, which is correctly shared between $U_{i}$ and GWN.

## 6. Performance Analysis of the Proposed Scheme

In this section, we summarize the performance analysis of our proposed scheme in terms of the computation and communication complexities. These two factors are the most important when measuring the performance of any user authentication and key agreement protocol for WSN, and it would be more efficient for the complexities to be less than that of existing schemes. We thus present a performance evaluation to compare our proposed scheme to other related schemes [13,14,15,16,17,18,19,20,28].

### 6.1. Computational Performance Analysis

In this subsection, we present a comparison of the computational costs, and measure the execution time. The computational analysis of an authentication protocol is generally conducted by focusing on operations performed by each party within the protocols. Therefore, for analysis of the computational costs, we concentrated on the operations that are conducted by the parties in WSNs: namely a user, a gateway node, a sensor node, and a base station. A base station is used to gather the information detected by sensor node or gateway node. Our scheme also analyzes the messages which are delivered in each communication party within the protocols. This analysis of the message size is relevant to the communication cost, and there are more details in Section 6.2. In order to facilitate the analysis of the computational costs, we define the following notation.

- $T_{H}$: the time to execute a one-way hashing operation
- $T_{E/D}$: the time to compute a symmetric-key encryption/decryption
- $T_{ECC}$: the time to compute an encryption/decryption operation in ECC-160 algorithm

In addition, in order to achieve accurate measurement, we performed an experiment. This experiment was performed using the Crypto++ Library [42] on a system using the 64-bits Windows 7 operating system, 3.2 GHz processor, 4 GB memory, Visual C++ 2013 Software, the SHA-1 hash function, the AES symmetric encryption/decryption function, and the ECC-160 function. According to our experiment, $T_{H}$ is nearly 0.0002 s on average, $T_{E/D}$ is nearly 0.0087 s on average and $T_{ECC}$ is nearly 0.6 s on average.

Table 3 compiles a comparative analysis of the computational cost among the related schemes [13,14,15,16,17,18,19,20,28]. For example to calculate computational costs, the computation costs of sensor node are $3T_{H}$ from our proposed scheme in Table 3. Sensor node is the sum of three values from hash operation, $SK=h(DID_{i}||h(x_{s}||SID_{n})||R_{2}\left|\right|T_{2})$, $B_{i}^{*}=h(DID_{i}||SK||h(x_{s}||SID_{n})||SID_{n}\left|\right|T_{2})$, and $C_{i}=h(h(x_{s}||SID_{n})||SK||DID_{i}||SID_{n}\left|\right|T_{3})$, in login and verification phase. However, the value of $h(x_{s}||SID_{n})$ is not counted, since it is already contained in sensor node. Using this computation method, we analyze by comparing the computational load during the login and verification phases. Table 3 shows that Yeh et al.’s scheme [18] imposes the highest computational load, because their scheme uses an ECC operation. In contrast with Chen et al.’s scheme [28], the total computational costs for the proposed scheme uses only three more hash operations. However, there is almost no difference between them in terms of computational complexities, because the hash function is an extremely lightweight operation. In addition, even though our proposed scheme is more computationally costly than some of the other schemes, this should be easily tolerated because our proposed scheme assures higher security, and affords resistance to most well known attacks, while providing functionality.

Table 4 presents the time consumption of the proposed scheme and the other related schemes [13,14,15,16,17,18,19,20,28]. Most of authentication researches [24,29,43,44,45] use the following ways to compute execution time of protocol: (1) calculate protocol’s computational costs, (2) measure each operation’s execution time by simulation, and (3) apply the execution time derived by (2) into (1). The values on Table 4 are also based on total computational costs derived by Table 3. That is, the values of simulations ($T_{H}$≈ 0.0002, $T_{E/D}$≈ 0.0087, $T_{ECC}$≈ 0.6) are substituted into the total computational costs on Table 3. Total computational costs of proposed scheme are $13T_{H}+4T_{E/D}$, which is (13×0.002+4×0.0087≈0.0374 s). Other scheme’s execution times are compared in the same way: Das et al. [13] ($9T_{H}\approx 9\times 0.0002$), K-A- [14] ($12T_{H}\approx 12\times 0.0002$), Vaidya et al. [15] ($13T_{H}\approx 13\times 0.0002$), C-S- [16] ($10T_{H}\approx 10\times 0.0002$), Fan et al. [17] ($19T_{H}\approx 19\times 0.0002$), Yeh et al. [18] ($8T_{H}+6T_{ECC}\approx 8\times 0.0002+6\times 0.6$), Das et al. [19] ($10T_{H}+6T_{E/D}\approx 10\times 0.0002+6\times 0.0087$), Xue et al. [20] ($26T_{H}\approx 26\times 0.0002$), Chen et al. [28] ($10T_{H}+4T_{E/D}\approx 10\times 0.0002+4\times 0.0087$) are 0.0018 s, 0.0024 s, 0.0026 s, 0.002 s, 0.0038 s, 3.6016 s, 0.0542 s, 0.0052 s, 0.0368 s, respectively. Table 4 shows that the execution time of our proposed scheme is only 0.0374 s, so it can be regarded as of negligible significance. Whereas, Yeh et al.’s scheme [18] using ECC operation requires 3.6016 s, and therefore Yeh et al.’s scheme turns out to be ineffective. There is no need for concern about the execution time difference between our scheme and the other systems. The Table 4 shows our scheme takes slightly more time, but it is hard for the users to perceive this time difference. From Table 3 and Table 4, we conclude that our proposed scheme considers the efficiency.

### 6.2. Communication Performance Analysis

In this subsection, we analyze the messages that are delivered to each party within the protocols. This analysis of the message size is relevant to the communication cost. We compare the number of messages and the total number of bytes for all messages to be transmitted during the login and verification phases. Table 5 shows the communication cost between our proposed scheme and the other schemes [13,14,15,16,17,18,19,20,28]. We have analyzed all the schemes mentioned in Table 5, and the details of algorithms of related works [13,14,15,16,17,18,19,20] are described in Appendix A, Appendix B, Appendix C, Appendix D, Appendix E, Appendix F, Appendix G and Appendix H. Based on [23], we set that both the block size of the symmetric encryption and one-way hash function h(·) are 20 bytes long, the identity $ID_{i}$ and password $PW_{i}$ are 8 bytes, the random number *b*, $R_{1}$, and $R_{2}$ are 16 bytes, the timestamp $T_{1}$–$T_{4}$ are 19 bytes, and ECC function is 15 bytes long. Table 5 shows that in Chen et al.’s scheme [28], the login request message $\langle ID_{i},A_{i},T_{1}\rangle$ requires (8 + 20 + 19) = 47 bytes, and the authentication message $\langle ID_{i},B_{i},T_{2}\rangle$ requires (8 + 20 + 19) = 47 bytes. The last two authentication messages $\langle C_{i},T_{3}\rangle$ and $\langle D_{i},T_{4}\rangle$ require (20 + 19) = 39 bytes and (20 + 19) = 39 bytes, respectively. Thus, their scheme requires a total of 172 bytes.

In our proposed scheme, the login request message $\langle DID_{i},A_{i},T_{1}\rangle$ requires (20 + 20 + 19) = 59 bytes, and the authentication message $\langle M_{i},DID_{i},B_{i},T_{2}\rangle$ requires (20 + 20 + 20 + 19) = 79 bytes. The second authentication message $\langle C_{i},T_{3}\rangle$ requires (20 + 19) = 39 bytes, and the third authentication message $\langle D_{i},T_{4}\rangle$ requires (20 + 19) = 39 bytes. Adding all these together, the communication overhead becomes (59 + 79 + 39 + 39) = 216 bytes. Table 5 shows that our proposed scheme requires a little more communication cost than Chen et al.’s scheme [28]. However, our scheme corrects the flaws of Chen et al.’s scheme, such as smart card loss attack, and denial of service attack. Also, even though our scheme requires a little more communication cost than some of the other schemes, we consider this acceptable because our proposed scheme assures security and provides additional functionalities, as Table 2 shows.

## 7. Conclusions

In this study, we analyze the security weaknesses of Chen et al.’s scheme, and show that their scheme is susceptible to smart card loss attack and denial of service attack. In addition, we also show that Chen et al.’s scheme cannot preserve user anonymity, and their scheme cannot quickly detect an incorrect password during the login phase. So, we propose a security enhanced user authentication and key agreement scheme using a symmetric cryptosystem for WSNs. The proposed scheme not only preserves the merits of Chen et al.’s scheme, but also fixes its security flaws. Our security and performance comparison shows that our protocol achieves both stronger security and higher efficiency. Therefore, we estimate that our proposed scheme is more suitable for applications in WSNs.

## Figures and Tables

**Table 1:** Notations.

Value	Description
$U_{i}$	Remote user
$S_{n}$	Sensor node
GWN	Gateway node
$ID_{i}$, $PW_{i}$	Identity and password of $U_{i}$
$SID_{n}$	Identity of $S_{n}$
$DID_{i}$	Dynamic identity of $U_{i}$
*k*	The symmetric key
$E_{k},D_{k}$	Encryption/Decryption with the symmetric key *k*
$x_{a}$	The secret parameter generated by the GWN, $\left(U_{i}\overset{x_{a}}{⟷}GWN\right)$
$x_{s}$	The shared key between the GWN and $S_{n}$
$h(x_{s}||SID_{n})$	The secret key instead of $x_{s}$ stored in $S_{n}$, $\left(GWN\overset{h(x_{s}||SID_{n})}{⟷}S_{n}\right)$
*b*	A random number chosen by $U_{i}$
$R_{i}$	Cryptographic random numbers or nonces
h(·)	One-way hash function
X||Y	Concatenate operation
⊕	XOR operation
$T_{1}$,$T_{2}$,$T_{3}$,$T_{4}$	Current timestamp
SK	Session key
ΔT	The maximum of transmission delay time

**Table 2:** Security comparison of our proposed scheme and other related schemes.

Features	Das et al. [13]	K-A- [14]	Vaidya et al. [15]	C-S- [16]	Fan et al. [17]	Yeh et al. [18]	Das et al. [19]	Xue et al. [20]	Chen et al. [28]	Proposed Scheme
Proposition 1	×	√	×	√	√	×	×	×	×	√
Proposition 2	√	×	×	×	×	√	√	×	√	√
Proposition 3	×	×	×	×	√	√	√	√	√	√
Proposition 4	×	×	×	×	√	×	√	×	×	√
Proposition 5	×	√	×	×	√	×	×	×	√	√
Proposition 6	×	×	×	×	×	×	×	×	√	√
Proposition 7	√	√	√	×	√	√	√	√	×	√
Proposition 8	√	√	√	√	√	×	√	√	√	√
Proposition 9	×	√	√	×	√	√	×	×	√	√
Proposition 10	√	×	√	×	√	√	√	√	×	√
Proposition 11	√	√	√	√	√	√	√	√	√	√
Proposition 12	√	√	√	√	√	√	√	√	√	√
Proposition 13	×	√	√	×	×	×	√	√	√	√
Proposition 14	×	×	√	×	√	√	√	√	√	√

**Table 3:** Comparison of the computational cost between our proposed scheme and other related schemes.

Schemes	User	Gateway Node	Sensor Node	Base Station	Total
Proposed scheme	$5T_{H}+2T_{E/D}$	$5T_{H}+2T_{E/D}$	$3T_{H}$	-	$13T_{H}+4T_{E/D}$
Chen et al. [28]	$2T_{H}+2T_{E/D}$	$5T_{H}+2T_{E/D}$	$3T_{H}$	-	$10T_{H}+4T_{E/D}$
Xue et al. [20]	$7T_{H}$	$13T_{H}$	$6T_{H}$	-	$26T_{H}$
Das et al. [19]	$5T_{H}+1T_{E/D}$	$2T_{H}+2T_{E/D}$	-	$3T_{H}+3T_{E/D}$	$10T_{H}+6T_{E/D}$
Yeh et al. [18]	$1T_{H}+2T_{ECC}$	$4T_{H}+2T_{ECC}$	$3T_{H}+2T_{ECC}$	-	$8T_{H}+6T_{ECC}$
Fan et al. [17]	$7T_{H}$	$8T_{H}$	$2T_{H}$	$2T_{H}$	$19T_{H}$
C-S- [16]	$4T_{H}$	$5T_{H}$	$1T_{H}$	-	$10T_{H}$
Vaidya et al. [15]	$6T_{H}$	$5T_{H}$	$2T_{H}$	-	$13T_{H}$
K-A- [14]	$4T_{H}$	$6T_{H}$	$2T_{H}$	-	$12T_{H}$
Das et al. [13]	$4T_{H}$	$1T_{H}$	$4T_{H}$	-	$9T_{H}$

**Table 4:** Comparison of the execution times.

Das’s [13]	K-A-’s [14]	Vaidya’s [15]	C-S-’s [16]	Fan’s [17]	Yeh’s [18]	Das’s [19]	Xue’s [20]	Chen’s [28]	Proposed Scheme
≈0.0018 s	≈0.0024 s	≈0.0026 s	≈0.002 s	≈0.0038 s	≈3.6016 s	≈0.0542 s	≈0.0052 s	≈0.0368 s	≈0.0374 s

**Table 5:** Comparison of the communication cost between our proposed scheme and other related schemes.

Schemes	Total Number of Messages Required	Total Number of Bytes Required
Proposed scheme	4 Messages	216 Bytes
Chen et al. [28]	4 Messages	172 Bytes
Xue et al. [20]	6 Messages	284 Bytes
Das et al. [19]	4 Messages	253 Bytes
Yeh et al. [18]	3 Messages	118 Bytes
Fan et al. [17]	3 Messages	126 Bytes
Chen and Shih [16]	4 Messages	170 Bytes
Vaidya et al. [15]	5 Messages	157 Bytes
Khan and Alghathbar [14]	4 Messages	157 Bytes
Das et al. [13]	3 Messages	118 Bytes

**Figure A1:**
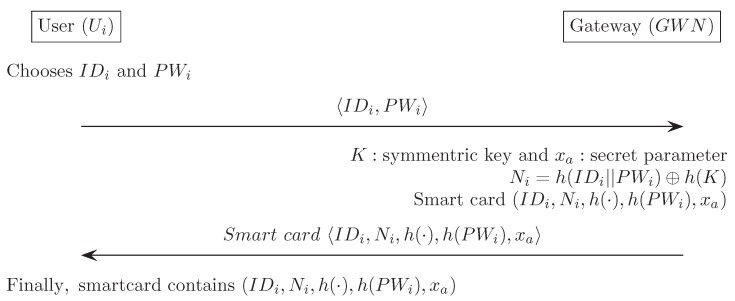
Registration phase for the Das et al.’s scheme [13].

**Figure A2:**
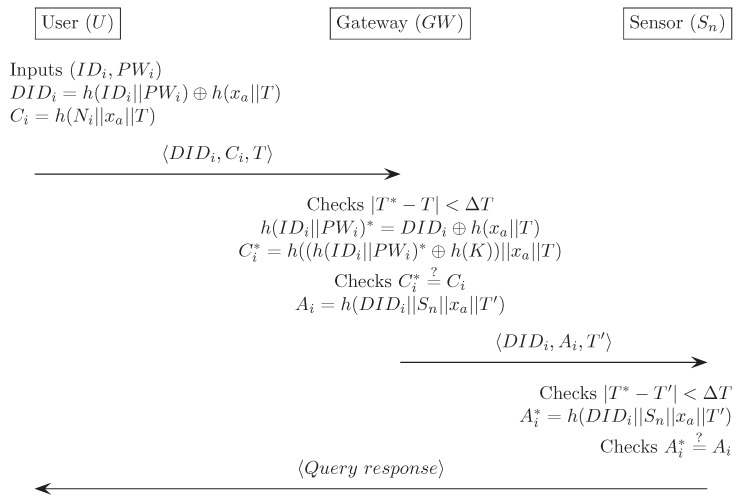
Login and verification phase for the Das et al.’s scheme [13].

**Figure B1:**
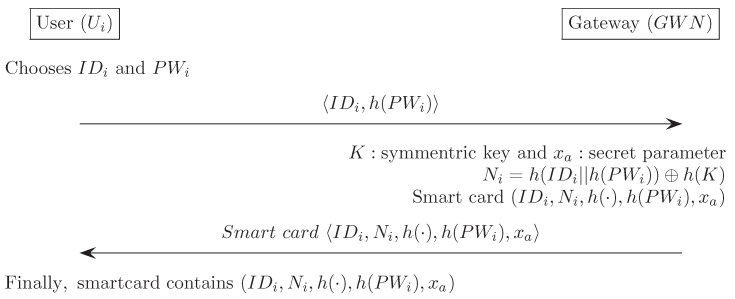
Registration phase for the Khan and Alghathbar’s scheme [14].

**Figure B2:**
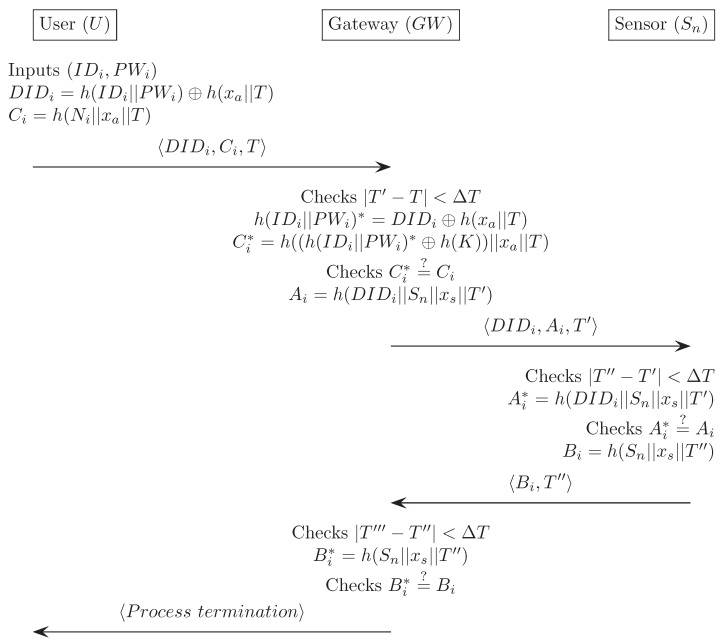
Login and verification phase for the Khan and Alghathbar’s scheme [14].

**Figure C1:**
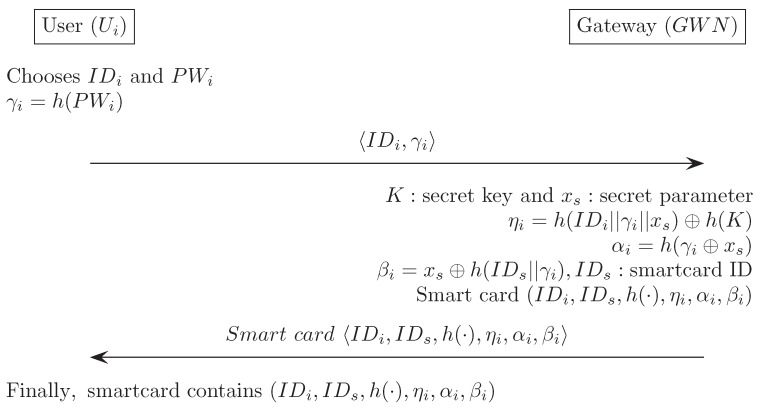
Registration phase for the Vaidya et al.’s scheme [15].

**Figure C2:**
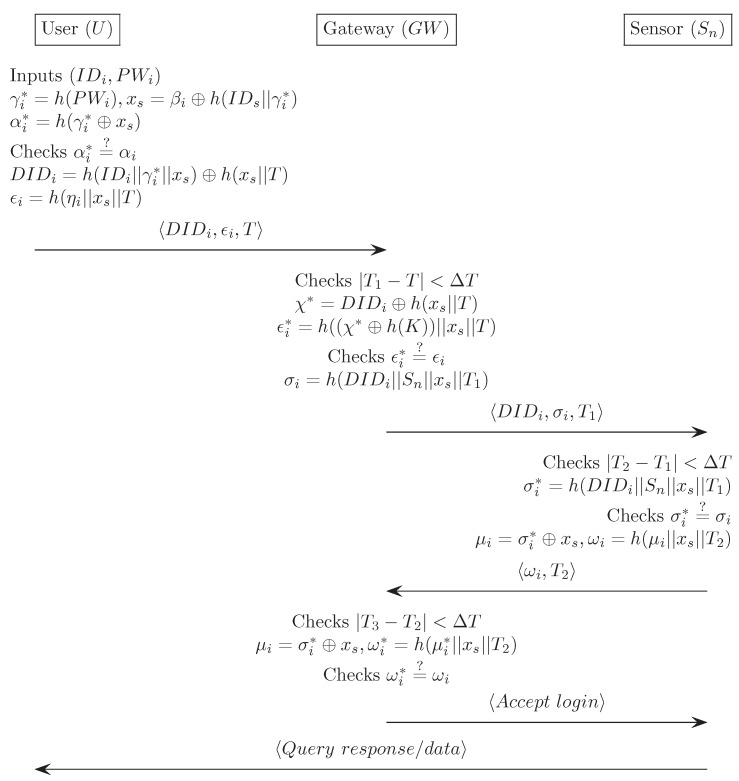
Login and verification phase for the Vaidya et al.’s scheme [15].

**Figure D1:**
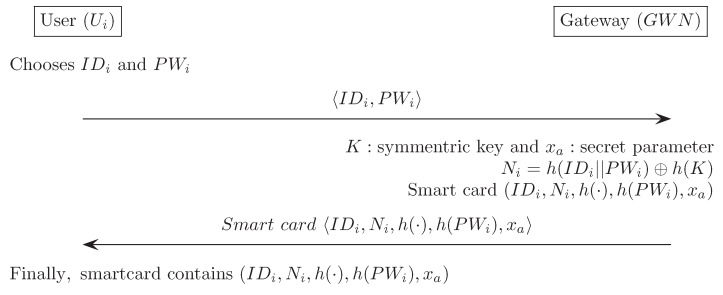
Registration phase for the Chen and Shih’s scheme [16].

**Figure D2:**
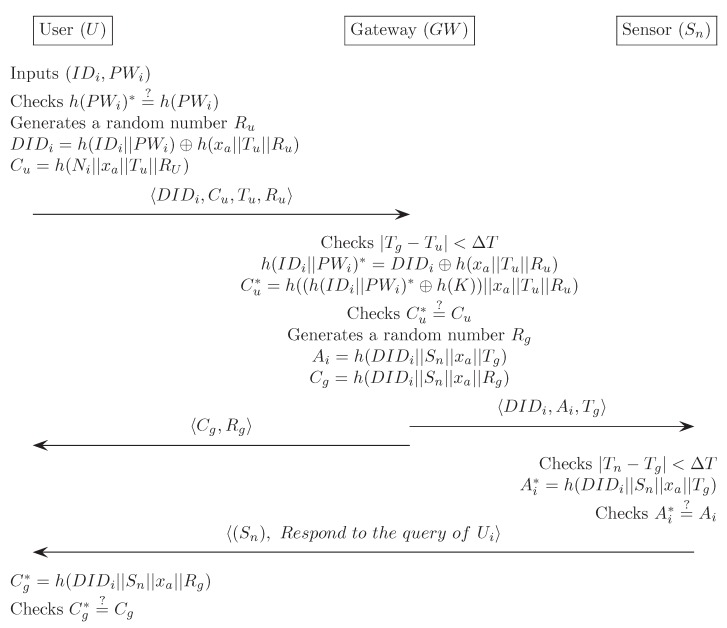
Login and verification phase for the Chen and Shih’s scheme [16].

**Figure E1:**
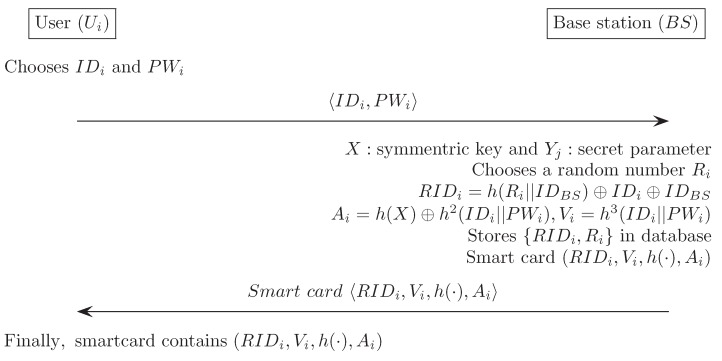
Registration phase for the Fan et al.’s scheme [17].

**Figure E2:**
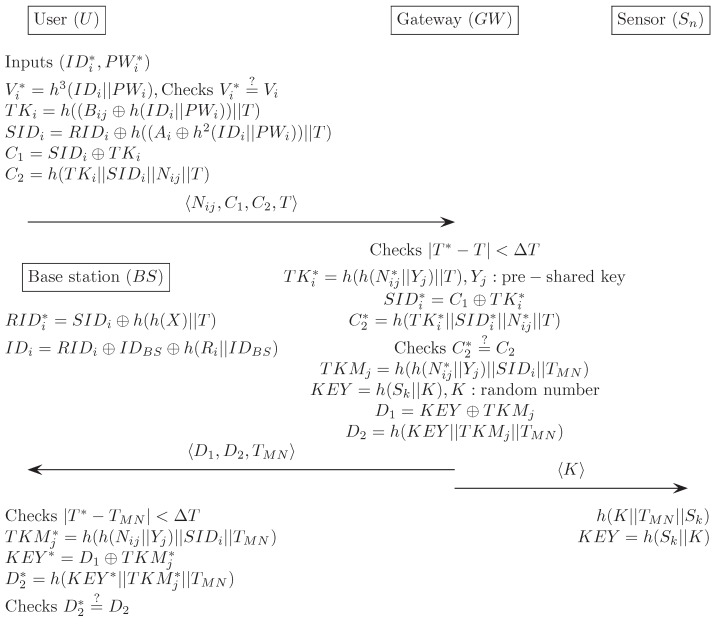
Login and verification phase for the Fan et al.’s scheme [17].

**Figure F1:**
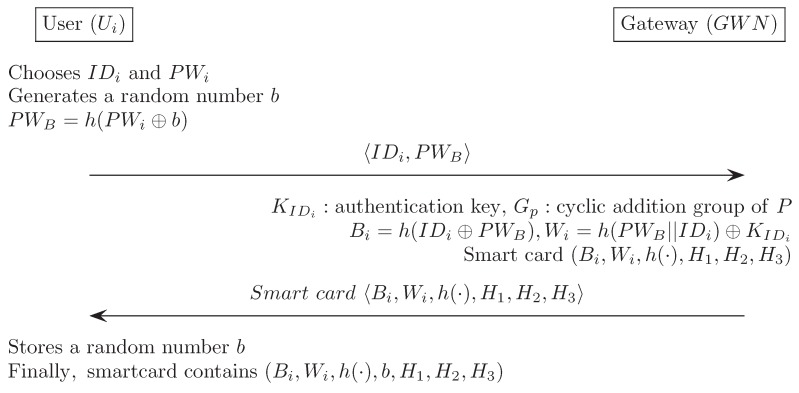
Registration phase for the Yeh et al.’s scheme [18].

**Figure F2:**
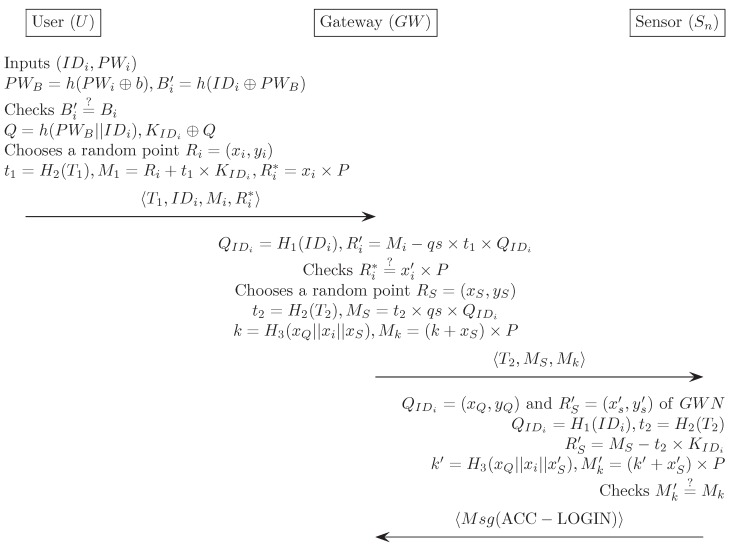
Login and verification phase for the Yeh et al.’s scheme [18].

**Figure G1:**
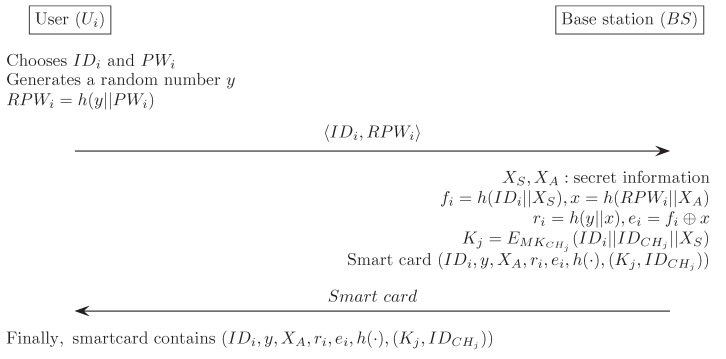
Registration phase for the Das et al.’s scheme [19].

**Figure G2:**
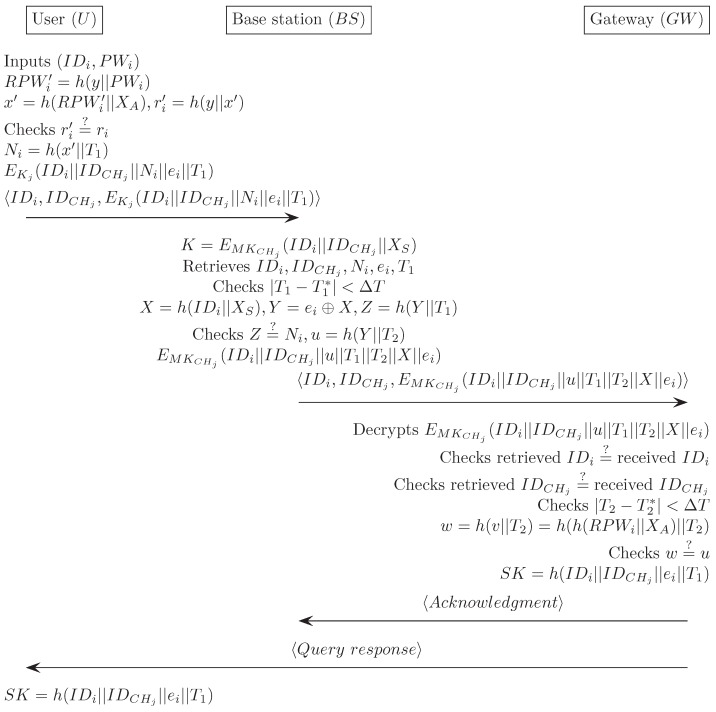
Login and verification phase for the Das et al.’s scheme [19].

**Figure H1:**
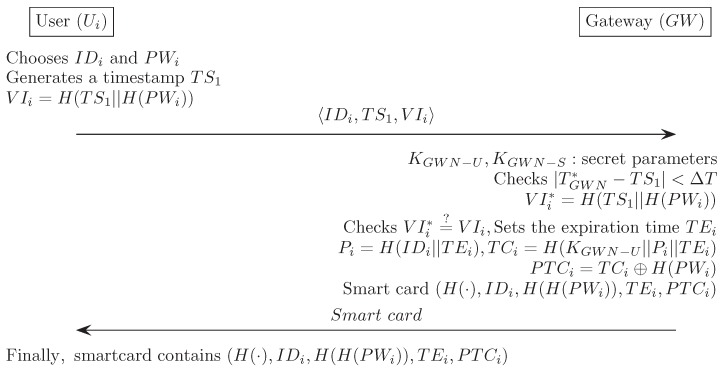
Registration phase for the Xue et al.’s scheme [20].

**Figure H2:**
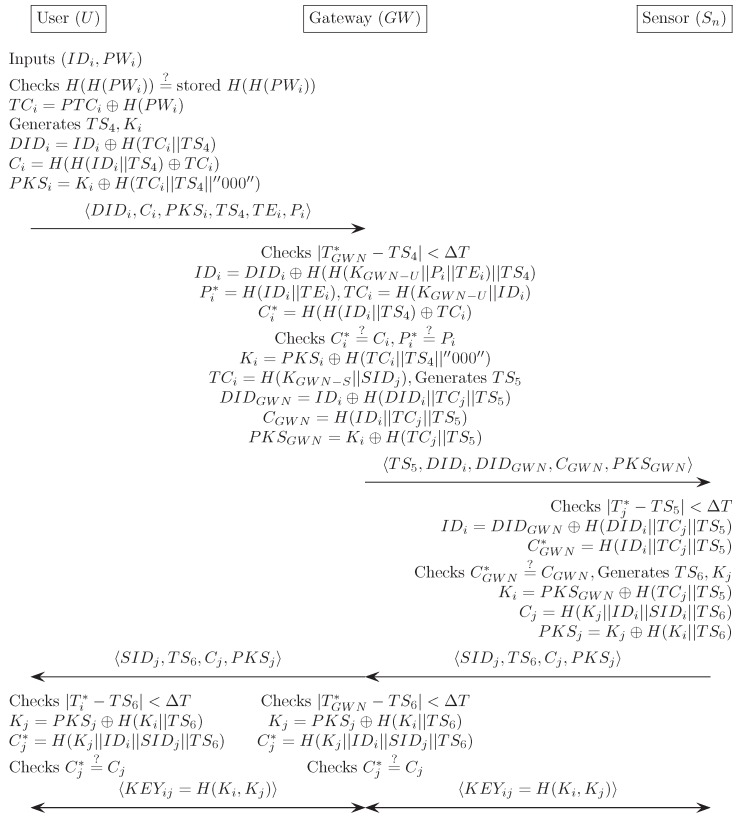
Login and verification phase for the Xue et al.’s scheme [20].

## Appendix A. Das et al.’s Authentication Scheme [13]

Das et al.’s authentication scheme is shown in Figure A1 and Figure A2.

## Appendix B. Khan and Alghathbar’s Authentication Scheme [14]

Khan and Alghathbar’s authentication scheme is shown in Figure B1 and Figure B2.

## Appendix C. Vaidya et al.’s Authentication Scheme [15]

Vaidya et al.’s authentication scheme is shown in Figure C1 and Figure C2.

## Appendix D. Chen and Shih’s Authentication Scheme [16]

Chen and Shih’s authentication scheme is shown in Figure D1 and Figure D2.

## Appendix E. Fan et al.’s Authentication Scheme [17]

Fan et al.’s authentication scheme is shown in Figure E1 and Figure E2.

## Appendix F. Yeh et al.’s Authentication Scheme [18]

Yeh et al.’s authentication scheme is shown in Figure F1 and Figure F2.

## Appendix G. Das et al.’s Authentication Scheme [19]

Das et al.’s authentication scheme is shown in Figure G1 and Figure G2.

## Appendix H. Xue et al.’s Authentication Scheme [20]

Xue et al.’s authentication scheme is shown in Figure H1 and Figure H2.
